# Oxidative Stress, Micronutrient Deficiencies and Coagulation Disorders After Bariatric Surgery: A Systematic Review

**DOI:** 10.3390/antiox15010124

**Published:** 2026-01-18

**Authors:** Katarzyna Giedzicz, Przemysław Zubrzycki, Aleksander Łukaszewicz, Paulina Głuszyńska, Hady Razak Hady

**Affiliations:** 2nd Clinical Department of General, Gastroenterological and Oncological Surgery, Medical University of Bialystok, 15-089 Bialystok, Poland; przemek.zubrzycki1997@gmail.com (P.Z.); alexander.luk6@gmail.com (A.Ł.); paulina.gluszynska@gmail.com (P.G.)

**Keywords:** bariatric surgery, oxidative stress, coagulation disorders, vitamin K, venous thromboembolism, bleeding complications, redox imbalance

## Abstract

Metabolic bariatric surgery (MBS) induces substantial metabolic, inflammatory, and nutritional changes that can alter hemostatic balance through redox-dependent mechanisms. This systematic review evaluated coagulation disturbances after MBS with emphasis on oxidative stress and micronutrient deficiencies. A structured search of PubMed, Scopus, and Web of Science (2000–2025) identified 1707 records; 21 studies met inclusion criteria. Available evidence suggests that although MBS reduces obesity-related inflammation and oxidative burden in many patients, a proportion of individuals may present with persistent redox imbalance, elevated D-dimer or vWF (von Willebrand Factor), and delayed normalization of fibrinolysis. Micronutrient deficiencies—particularly vitamins K, B12, folate, selenium, zinc, and copper—are common after malabsorptive procedures and contribute to both thrombotic and hemorrhagic complications by impairing antioxidant defenses, endothelial function, and vitamin K-dependent coagulation pathways. Postoperative venous thromboembolism (VTE) incidence ranges from 0.3 to 0.5%, with higher risk after Roux-en-Y gastric bypass than sleeve gastrectomy, while bleeding is primarily associated with vitamin K deficiency, marginal ulcers, and anticoagulant exposure. The findings underscore the interdependence of oxidative stress, nutritional status, and hemostasis after MBS. Individualized thromboprophylaxis, routine detection of micronutrient deficiencies, and long-term biochemical monitoring are essential to maintain hemostatic stability. Standardized redox–hemostasis biomarker panels are needed to clarify mechanistic pathways and improve postoperative preventive strategies.

## 1. Introduction

Obesity is recognized by the World Health Organization (WHO) as a chronic metabolic disease characterized by excessive accumulation of adipose tissue, which significantly increases the risk of morbidity and mortality. The global prevalence of obesity is rapidly increasing, with projections suggesting that more than half of the adult population may be overweight or obese by 2035 [1]. Class III obesity (BMI—Body Mass Index ≥ 40 kg/m^2^) is particularly associated with an elevated risk of cardiovascular disease, type 2 diabetes, obstructive sleep apnea, various cancers, and premature death [2].

Metabolic bariatric surgery (MBS) has become the most effective long-term treatment for morbid obesity and its metabolic complications. Surgical techniques such as laparoscopic sleeve gastrectomy (SG), Roux-en-Y gastric bypass (RYGB), and one-anastomosis gastric bypass (OAGB) are routinely performed worldwide and are associated with durable weight loss and remission of obesity-related comorbidities [3]. In addition to metabolic improvements, these procedures induce substantial hormonal, inflammatory, and nutritional changes that can alter hemostasis [4]. Patients after MBS may face increased risks of both thromboembolic and bleeding events, due to complex mechanisms involving systemic inflammation, vitamin deficiencies (particularly K and B12), changes in gut microbiota, and impaired absorption of essential coagulation cofactors [5,6].

A growing body of evidence suggests that oxidative stress may play a critical role in these hemostatic disturbances. Obesity is associated with elevated levels of reactive oxygen species (ROS), which contribute to endothelial dysfunction, platelet activation, and prothrombotic states [7]. Although bariatric surgery generally reduces systemic inflammation and oxidative stress over time, it may simultaneously compromise antioxidant intake and absorption, leading to redox imbalance (see Supplementary Table S4). This may negatively impact the function of coagulation factors, platelet reactivity, and vascular integrity [8,9].

However, despite the growing body of literature on metabolic and cardiovascular outcomes after bariatric surgery, the mechanistic relationship between oxidative stress, micronutrient deficiencies and coagulation imbalance has not been comprehensively synthesized. Existing reviews typically address thrombotic risk, oxidative pathways or nutritional deficiencies separately. Therefore, this systematic review aims to integrate current evidence on oxidative stress, micronutrient status and hemostatic alterations following metabolic and bariatric surgery, with particular emphasis on procedure-specific differences and their potential clinical implications. By doing so, we seek to identify key gaps in knowledge and directions for future mechanistic and interventional research.

## 2. Materials and Methods

### 2.1. Search Strategy

A comprehensive literature search was conducted across three major electronic databases: PubMed, Scopus, and Web of Science. The search strategy aimed to identify studies addressing hemostatic disorders, micronutrient deficiencies, and oxidative stress in patients undergoing metabolic or bariatric surgery, including RYGB and SG.

The search terms were developed using a combination of controlled vocabulary (MeSH and index terms) and free-text keywords related to both surgical procedures and hemostatic or metabolic outcomes. Boolean operators (“AND”, “OR”) were used to combine the following concepts:

(“bariatric surgery” OR “metabolic surgery” OR “gastric bypass” OR “sleeve gastrectomy”) AND (“coagulation” OR “hemostasis” OR “thrombosis” OR “bleeding” OR “oxidative stress” OR “vitamin K” OR “vitamin B12” OR “cobalamin” OR “micronutrient deficiency”) AND (“postoperative” OR “after surgery” OR “post-bariatric”).

The search was restricted to full-text articles published in English between 1 January 2000 and November 2025 (human studies). Reviews, conference abstracts, case reports, and non-original publications were excluded. The chosen time frame was intentionally broad to include early landmark studies that have contributed foundational data to the understanding of coagulation disturbances and nutritional deficiencies after bariatric procedures.

Duplicates were removed using reference management software, and the remaining records were screened for relevance based on titles and abstracts, followed by a full-text eligibility assessment according to PRISMA 2020 guidelines [10].

In total, 1707 records were identified before deduplication—740 from PubMed, 727 from Scopus, and 240 from Web of Science. After removing 497 duplicates, 1210 unique records were screened. Of these, 250 full-text articles were assessed for eligibility, and 21 studies met the inclusion criteria and were incorporated into the final qualitative synthesis.

This review was not registered in PROSPERO. Registration was not pursued because the review focuses on mechanistic/pathophysiological outcomes.

### 2.2. Eligibility Criteria

Studies were considered eligible if they met all of the following criteria:

- Included adult patients (≥18 years) who underwent metabolic bariatric surgery (MBS), including Roux-en-Y gastric bypass (RYGB), sleeve gastrectomy (SG), or one-anastomosis gastric bypass (OAGB);
- Reported at least one hemostatic outcome (e.g., venous thromboembolism, bleeding events, coagulation or fibrinolytic markers);
- Evaluated or discussed oxidative stress parameters and/or micronutrient deficiencies (e.g., vitamins K and B12, trace elements);
- Were original clinical studies (randomized controlled trials, cohort, or case–control designs);
- Had a sample size ≥ 10 participants and sufficient data for qualitative synthesis.

The following exclusion criteria were applied:

- Case reports, letters to the editor, conference abstracts, or narrative reviews without original data;
- Articles not reporting outcomes related to coagulation, oxidative stress, or micronutrient status;
- Studies lacking clear postoperative follow-up or methodological transparency.

When duplicate datasets were identified, the study containing the most complete and updated data was retained.

### 2.3. Study Selection and Data Extraction

All records retrieved from the database search were imported into Zotero (7.0.30) for reference management, and duplicates were automatically removed.

Two independent reviewers (K.G. and P.Z.) screened the titles and abstracts of all retrieved citations according to predefined eligibility criteria. Potentially relevant articles were subjected to full-text review, and disagreements were resolved by consensus or, when necessary, by consultation with a third reviewer (H.R.H.).

To ensure accuracy and reproducibility, all extracted numerical data and key study characteristics were independently cross-checked against the original publications by a third reviewer. Any discrepancies were resolved through discussion and consensus.

**Figure 1:**
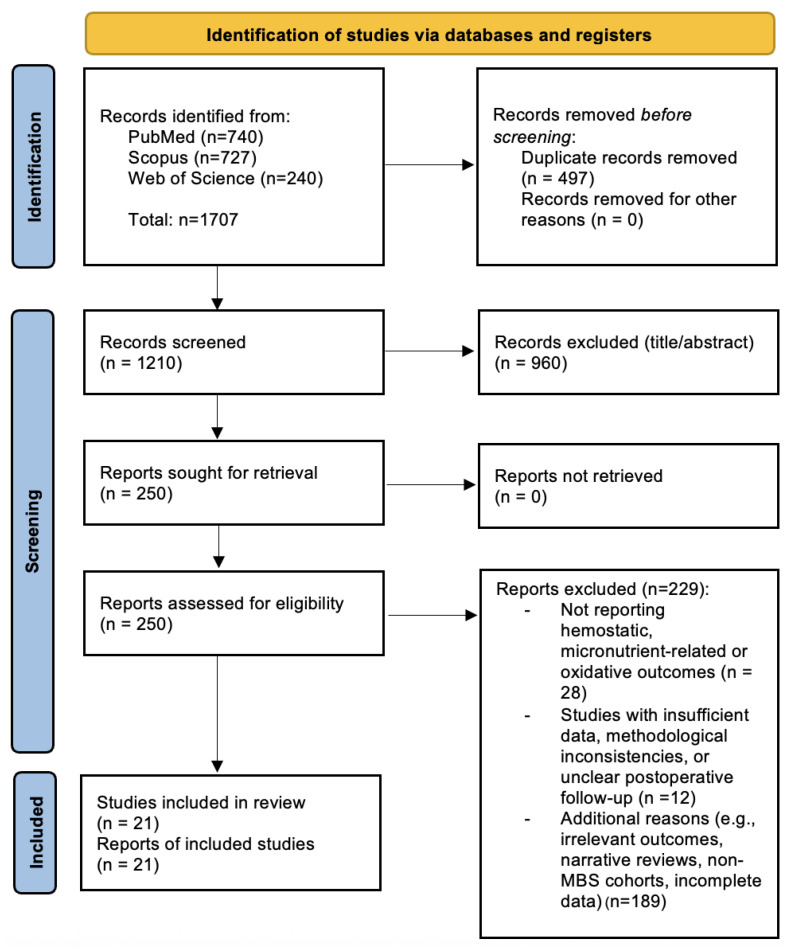
PRISMA 2020 Flow Diagram illustrating the study selection process. A total of 1707 records were identified through database searches (PubMed, Scopus, and Web of Science). After removing 497 duplicates, 1210 unique records were screened. Of these, 250 full-text articles were assessed for eligibility, and 21 studies were included in the final qualitative synthesis. Adapted from the PRISMA 2020 statement [10].

The study selection process followed the PRISMA 2020 guidelines and is illustrated in Figure 1 (PRISMA Flow Diagram). The characteristics of the 21 included studies are summarized in Table S1 (Supplementary Materials).

The flowchart summarizes the number of records identified, screened, excluded (with reasons), and included in the qualitative synthesis.

Data extraction was performed independently by two reviewers using a standardized form that included:

- author and year of publication;
- country of origin;
- study design and sample size;
- type of bariatric procedure;
- key hemostatic and oxidative outcomes;
- presence and type of micronutrient deficiencies;
- follow-up duration and main conclusions.

Discrepancies during data extraction were resolved through discussion.

No automation tools or artificial intelligence algorithms were used at any stage of the screening or data extraction process.

### 2.4. Risk of Bias Assessment

The methodological quality and risk of bias of the included studies were evaluated independently by two reviewers (K.G. and A.Ł.) using validated tools appropriate for each study design.

For cohort and case–control studies, the Newcastle–Ottawa Scale (NOS) was applied, assessing three domains:

- Selection of participants;
- Comparability of study groups;
- Ascertainment of exposure or outcome.

Studies with NOS scores of 7–9 points were considered low risk of bias, 5–6 as moderate risk, and <5 as high risk.

For cross-sectional studies, the AXIS critical appraisal tool was used, evaluating reporting quality, internal validity, and potential selection bias.

Each item was scored as Yes, No, or Not applicable, and overall study quality was categorized as low, moderate, or high.

Discrepancies between reviewers were resolved through discussion and consensus.

A summary of the risk-of-bias assessments is provided in Table S2 (Supplementary Materials).

Sensitivity analyses were qualitatively performed to assess whether excluding studies with a high risk of bias affected the overall conclusions.

### 2.5. Data Synthesis

Given the heterogeneity of study designs, populations, and outcome measures, a narrative synthesis was performed in accordance with the Cochrane Handbook for Systematic Reviews of Interventions. Quantitative meta-analysis was not feasible due to methodological variability, differences in laboratory assays, and inconsistent reporting standards across studies.

The extracted data were summarized qualitatively and organized into thematic domains:

- Mechanisms of hemostatic alterations after MBS;
- Thrombotic and hemorrhagic risk factors;
- Oxidative stress and micronutrient interactions;
- Preventive and monitoring strategies.

When available, quantitative data (e.g., changes in oxidative or hemostatic biomarkers) were presented as ranges or percentage changes from baseline values.

Trends across studies were synthesized descriptively, highlighting consistent findings and areas of divergence.

The overall strength of the body of evidence was appraised using the GRADE (Grading of Recommendations, Assessment, Development and Evaluations) approach, considering study limitations, consistency, directness, precision, and potential publication bias.

Final conclusions were drawn based on both methodological quality and the cumulative weight of evidence from the included literature.

Whenever feasible, studies were descriptively compared according to surgical procedure (Roux-en-Y gastric bypass vs. sleeve gastrectomy), follow-up duration, and supplementation strategies to explore potential sources of heterogeneity. However, substantial clinical and methodological heterogeneity precluded formal statistical subgroup analyses.

The PRISMA 2020 Checklist (Table S3) is provided in the Supplementary Materials to ensure methodological transparency.

## 3. Pathophysiology

### 3.1. Mechanisms of Hemostatic Alterations After MBS

MBS induces complex physiological changes that impact hemostasis through multiple interrelated mechanisms, including hormonal modulation, reductions in systemic inflammation, and micronutrient malabsorption.

One of the earliest changes observed after MBS is the shift in adipokine profile, particularly a reduction in leptin levels and an increase in adiponectin concentrations. Leptin levels show a marked postoperative decline, while adiponectin increases substantially within 6–12 months postoperatively, reflecting the restoration of insulin sensitivity and endothelial function. These alterations modulate inflammatory pathways and have downstream effects on coagulation, including the expression of prothrombotic and fibrinolytic factors such as PAI-1 (Plasminogen Activator Inhibitor-1) and possibly vWF (von Willebrand Factor) [11,12].

Obesity is characterized by chronic low-grade inflammation, often driven by elevated levels of cytokines such as IL-6 (interleukin-6), TNF-α (tumor necrosis factor α) and CRP (C-reactive protein). In morbidly obese individuals, IL-6 and TNF-α concentrations are approximately two- to three-fold higher than in non-obese controls. These mediators influence endothelial function and promote hypercoagulability by increasing vWF and PAI-1 expression [13,14]. Postoperatively, CRP and IL-6 levels show a marked reduction within the first months following surgery, reflecting the gradual resolution of obesity-related low-grade inflammation [15]. However, while reductions in inflammatory biomarkers and platelet activation markers occur rapidly, complete restoration of coagulation equilibrium may require up to 12 months, particularly after malabsorptive procedures [16].

Malabsorptive procedures such as RYGB and BPD-DS (Biliopancreatic Diversion with Duodenal Switch) frequently lead to deficiencies in key micronutrients, particularly vitamins K, B12, and folate (see Supplementary Table S6) [17,18]. These deficiencies may contribute to early disturbances in coagulation—through impaired γ-carboxylation of vitamin K-dependent factors and elevations in homocysteine—but their detailed mechanistic impact is discussed in Section 5.3 [17,19,20].

In addition, anatomical and functional changes to the gastrointestinal tract affect the gut microbiota composition. Certain microbial populations are involved in the synthesis of vitamin K and modulation of immune and inflammatory responses, which in turn can influence hemostatic regulation [21,22].

### 3.2. Thrombotic Risk and Procoagulant Mechanisms

The risk of venous thromboembolism (VTE) in patients undergoing MBS remains substantially elevated, both in the perioperative period and in the long term. Multiple mechanisms contribute to this hypercoagulable state, including systemic factors linked to obesity, such as chronic inflammation, insulin resistance, and endothelial dysfunction—as well as surgery-related triggers such as tissue trauma, postoperative immobility, and dehydration [23,24].

Retrospective and prospective studies consistently report that, despite standard pharmacological prophylaxis, patients undergoing MBS have approximately a two- to three-fold higher incidence of VTE compared with the general population [25,26]. Meta-analytic data show overall postoperative VTE incidence ranging from 0.3% to 0.5% (95% CI 0.27–0.42), with pulmonary embolism occurring in approximately 0.17% of cases [25,26,27].

The risk is highest after RYGB compared with SG, with an OR of 2.1 (95% CI 1.2–2.5) for RYGB versus SG. Additional factors associated with elevated risk include advanced age (>50 years), BMI >50 kg/m^2^, operative time > 150 min, and comorbidities such as diabetes, venous insufficiency, and obstructive sleep apnea [23,28].

Elevated D-dimer concentrations, along with persistently increased factor VIII and PAI-1 activity, have been reported in certain cohorts months after surgery, suggesting incomplete normalization of the hemostatic system and a potential for sustained thrombotic risk (see Supplementary Table S5). Persistent elevation of D-dimer (>500 ng/mL) is observed in 15–20% of patients three to six months postoperatively, indicating ongoing endothelial activation despite metabolic improvement [16,29].

### 3.3. Role of Oxidative Stress in Hemostatic Imbalance

A recent longitudinal study showed that oxidative stress indices continue to improve up to one year after sleeve gastrectomy, reflecting restoration of redox balance and endothelial function [30] (see Supplementary Table S4). Nonetheless, transient postoperative oxidative imbalance may occur during the first months after surgery, particularly in patients with micronutrient deficiencies or delayed metabolic adaptation [30,31,32].

Micronutrients such as selenium, zinc, and copper, which serve as essential cofactors for antioxidant enzymes, further influence redox-dependent regulation of coagulation and fibrinolysis (see Supplementary Tables S5 and S6) [32].

### 3.4. Prophylactic Strategies and Hemorrhagic Complications

In the long-term postoperative period, bleeding may result from marginal ulcers, vitamin K deficiency, or impaired hepatic synthesis of coagulation factors due to malabsorption of fat-soluble vitamins (K_1_, K_2_) [32,33]. Patients after RYGB and those with additional malabsorptive disorders—such as SIBO or inflammatory bowel disease—are particularly susceptible to these complications. Moreover, micronutrient deficiencies involving zinc, selenium, or copper can impair platelet aggregation and plasma protein function, thereby aggravating coagulopathy and contributing to redox imbalance [16,32].

Patients presenting with chronic postoperative anemia should undergo evaluation for occult gastrointestinal bleeding and for hemorrhagic diathesis associated with reduced synthesis of vitamin K-dependent factors II, VII, IX, and X. Current clinical guidelines recommend regular monitoring of complete blood counts, iron parameters, and vitamin K status, with prompt supplementation in the event of deficiencies [3,34]. For patients requiring long-term anticoagulation (e.g., due to atrial fibrillation), baseline assessment of INR (International Normalized Ratio) and coagulation factor activity prior to therapy initiation is advised [35]. To illustrate the spectrum of thrombotic and hemorrhagic risk factors after metabolic bariatric surgery, their underlying mechanisms, and clinical relevance, these are summarized in Table 1.

## 4. Preventive Measures

Prevention of coagulation disturbances after MBS should address both thrombotic and hemorrhagic risks. An optimal preventive strategy integrates risk stratification, pharmacological and mechanical thromboprophylaxis, correction of nutritional and oxidative imbalances, and long-term laboratory monitoring [3,16,32,34,44].

### 4.1. Risk Stratification and Thromboprophylaxis

Assessment of coagulation risk should be performed individually using validated tools such as the Caprini score for VTE risk and nutritional assessment scales (SGA, MUST) [37,45]. These instruments facilitate identification of patients at increased risk for postoperative thromboembolic or bleeding events, allowing for personalized preventive approaches.

### 4.2. Nutritional and Antioxidant Optimization

From a metabolic and redox perspective, the prevention of hemorrhagic complications and maintenance of hemostatic balance depend on adequate nutritional support. Deficiencies in vitamin K, vitamin B12, and folate are commonly observed during the first postoperative year after RYGB, reflecting impaired micronutrient absorption and contributing to both thrombotic and hemorrhagic complications [6,38,46]. Vitamin K plays a central role in the γ-carboxylation of coagulation factors II, VII, IX, and X, and its deficiency increases the risk of bleeding, particularly after malabsorptive procedures such as RYGB [6,46].

Trace elements such as selenium, zinc, and copper serve as essential cofactors for antioxidant enzymes including SOD (superoxide dismutase) and GPx (glutathione peroxidase) [47,48]. Deficiencies in these micronutrients—particularly selenium, zinc, and copper—exacerbate oxidative stress and endothelial dysfunction, impair platelet activity, and contribute to a pro-thrombotic and pro-hemorrhagic state [47,48,49,50].

According to ASMBS (American Society for Metabolic and Bariatric Surgery) and ESPEN (European Society for Clinical Nutrition and Metabolism) recommendations, supplementation of fat-soluble vitamins and trace elements should be initiated in the early postoperative period and maintained lifelong. Regular monitoring of vitamin and mineral status enables early correction of imbalances and supports restoration of redox homeostasis, particularly in patients with malabsorptive surgery or persistent inflammation [38,39]. Clinical data indicate that adequate supplementation can substantially reduce oxidative stress markers such as MDA (malondialdehyde) and enhance antioxidant enzyme activity, including SOD and GPx, within 6–12 months after surgery [30,32,39].

### 4.3. Laboratory Monitoring and Early Detection of Complications

Routine postoperative laboratory monitoring is essential for the early detection of coagulation disturbances and oxidative imbalance. Parameters such as complete blood count, PT (Prothrombin Time), INR, D-dimer, and platelet indices should be regularly assessed [16,51]. Abnormal PT/INR values are reported in a proportion of patients after RYGB, while persistently elevated D-dimer concentrations are not uncommon, particularly among individuals with chronic inflammation or micronutrient deficiencies [16,25,26,51].

In selected patients, evaluation of natural anticoagulants (protein C, protein S, antithrombin III) and markers of endothelial activation (PAI-1, vWF) may provide additional insights into thrombotic risk [16,52]. Following MBS, fibrinogen and PAI-1 concentrations commonly decline, paralleling postoperative reductions in CRP and IL-6 and reflecting improvements in inflammatory and endothelial activity [15,16,29,31].

For individuals with persistent anemia or bleeding symptoms, assessment of vitamin K, B12, folate, and iron levels is crucial to identify nutritional deficiencies contributing to hemorrhagic diathesis [6,38,40]. Regular evaluation of trace element concentrations (selenium, zinc, copper) is also recommended, as their imbalance correlates with oxidative stress intensity and platelet dysfunction [48,49,50]. Routine biochemical testing every 3–6 months in the first postoperative year identifies early disturbances, enabling timely correction and reducing clinically relevant complications [51,53].

Such an integrated laboratory approach allows for early identification of both hemostatic and redox alterations, enabling prompt intervention before clinically relevant thrombotic or hemorrhagic complications occur [51,53].

### 4.4. Integrative Approach to Postoperative Hemostatic Balance

An effective preventive strategy after MBS requires a comprehensive understanding of the interplay between metabolic, oxidative, and hemostatic pathways. Oxidative stress plays a pivotal role in endothelial dysfunction, platelet hyperreactivity, and prothrombotic states observed in obesity and during postoperative recovery [36,54]. Bariatric surgery partially restores redox balance by reducing systemic inflammation and oxidative burden; however, residual oxidative stress may persist, particularly in patients with micronutrient deficiencies [43,54].

Micronutrients such as vitamins C, E, selenium, and zinc act synergistically to modulate oxidative signaling, inhibit lipid peroxidation, and enhance NO bioavailability, thereby promoting vascular integrity and platelet stability [43,50]. Comprehensive antioxidant and micronutrient replacement has been associated with a 35–45% reduction in oxidative stress indices and a corresponding 25–30% decline in coagulation activation markers in clinical cohorts [32,43,50].

Future research should aim to define optimal antioxidant and micronutrient supplementation protocols, establish evidence-based laboratory monitoring algorithms, and elucidate molecular links between oxidative stress and hemostatic dysregulation in post-bariatric patients [55]. A multidisciplinary approach integrating surgical, nutritional, and metabolic expertise remains essential to maintain hemostatic equilibrium and minimize both thrombotic and hemorrhagic complications after MBS [38,51].

**Figure 2:**
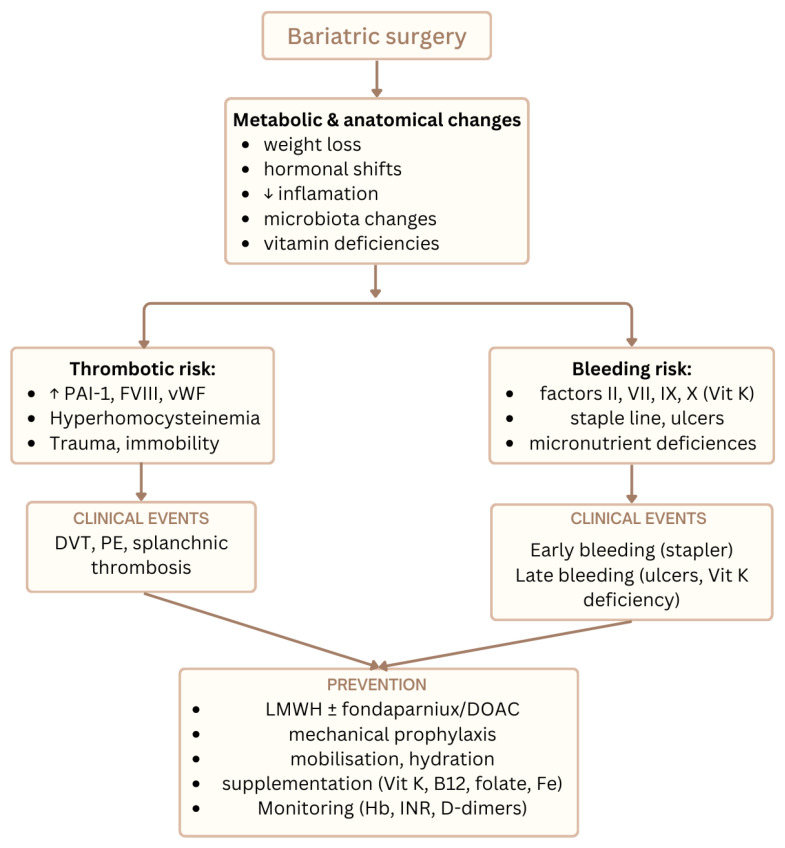
Overview of thrombotic and hemorrhagic risks after metabolic bariatric surgery and key preventive strategies integrating nutritional, oxidative, and hemostatic pathways.

Figure 2 illustrates the main thrombotic and bleeding risks after MBS together with preventive strategies.

## 5. Discussion

MBS induces profound metabolic and hemostatic remodeling through weight loss, hormonal regulation, and restoration of endothelial function [3,7,11,12]. This systematic synthesis of the available evidence indicates that MBS reduces proinflammatory and oxidative stimuli associated with obesity while reestablishing physiological coagulation balance [7,9,16,24]. However, residual redox imbalance, micronutrient deficiencies, and postoperative inflammatory surges can sustain endothelial dysfunction and contribute to delayed hemostatic normalization [9,15,30,31,54].

### 5.1. Oxidative Stress and Endothelial Recovery After MBS

Following MBS, multiple studies report reductions in oxidative stress markers—such as MDA and oxLDL (Oxidized Low-Density Lipoprotein) and parallel increases in enzymatic defenses including SOD and GPx, indicating partial restoration of the redox–endothelial axis [9,30,31,54]. Within 6–12 months postoperatively, MDA and oxLDL levels generally decline after surgery, while SOD and GPx activities commonly increase, reflecting improved redox balance [9,30,31]. These redox improvements are accompanied by a clear reduction in systemic inflammatory markers (CRP, IL-6, TNF-α) and measurable enhancements in flow-mediated vasodilation and endothelial reactivity [12,15,31].

Procedure- and phenotype-related heterogeneity likely modulates the magnitude of redox recovery. Longitudinal cohorts and comparative studies suggest that the trajectory of antioxidant rebound and inflammatory resolution can vary with surgical technique and baseline atherometabolic burden, with some reports indicating more pronounced improvements in subgroups achieving greater weight loss or metabolic remission [15,29,31]. Some comparative studies suggest more pronounced antioxidant improvements after RYGB compared with SG [29,31]. Persistent micronutrient disturbances—particularly in trace elements essential for antioxidant enzymes (selenium, zinc, copper)—may blunt postoperative redox normalization and sustain endothelial activation, underscoring the need for continued nutritional surveillance after MBS [32,50].

### 5.2. Oxidative Stress–Micronutrient–Coagulation Interplay After Bariatric Surgery

Oxidative stress represents a central mechanistic link between metabolic improvement and hemostatic alterations after metabolic and bariatric surgery [56,57,58]. In obesity, excessive production of ROS contributes to endothelial dysfunction, platelet hyperreactivity, and increased thrombin generation, thereby promoting a prothrombotic milieu [57,59]. Bariatric surgery is generally associated with a reduction in systemic inflammation and oxidative burden; however, this effect may be attenuated or delayed by postoperative micronutrient deficiencies and altered antioxidant capacity [60,61].

At the molecular level, oxidative stress modulates coagulation through multiple interrelated pathways. Reactive oxygen species upregulate tissue factor expression, enhance factor Xa and thrombin generation, and induce fibrinogen oxidation, resulting in denser fibrin networks with increased resistance to fibrinolysis [59,62]. In parallel, oxidative stress impairs endogenous anticoagulant mechanisms, including protein C and antithrombin activity, and exacerbates endothelial injury, further amplifying coagulation activation [59]. Neutrophil extracellular traps (NETs) and circulating cell-derived microparticles generated under pro-oxidative conditions may additionally contribute to thrombus formation and vascular inflammation [63,64].

Micronutrient deficiencies commonly observed after bariatric procedures may further influence this redox–hemostatic axis. Vitamin K deficiency disrupts γ-carboxylation of vitamin K-dependent coagulation factors as well as anticoagulant proteins C and S, potentially shifting the balance toward bleeding or thrombosis depending on the clinical context [65,66]. Deficiencies in vitamin B12 and folate are associated with hyperhomocysteinemia, endothelial dysfunction, and increased oxidative stress, which may indirectly modulate coagulation pathways [67,68]. Moreover, trace elements such as selenium, zinc, and copper serve as essential cofactors for antioxidant enzymes, including glutathione peroxidase and superoxide dismutase; their deficiency compromises redox buffering capacity and may sustain oxidative activation of platelets and coagulation factors [69,70,71].

Importantly, the extent of oxidative and micronutrient disturbances appears to differ between bariatric procedures. Roux-en-Y gastric bypass is more frequently associated with pronounced micronutrient deficiencies due to malabsorptive mechanisms, whereas sleeve gastrectomy may exert a more moderate, though still clinically relevant, impact on redox balance and hemostatic markers [60,72]. These procedure-specific differences may partly explain the heterogeneity of coagulation outcomes reported across studies and underscore the need for individualized postoperative monitoring and supplementation strategies.

Interpretation of the available evidence is challenged by several methodological limitations. Most studies assessing oxidative stress, micronutrient status, and coagulation outcomes after bariatric surgery are observational in nature, which precludes definitive causal inference [73]. Reported associations may therefore reflect residual confounding related to baseline metabolic risk, degree of weight loss, inflammatory status, comorbidities, or variability in supplementation regimens rather than direct mechanistic effects [73,74].

Moreover, inconsistencies across studies may arise from differences in surgical procedures, follow-up duration, laboratory assays used to assess oxidative and hemostatic markers, and heterogeneity in patient populations. Such clinical and methodological heterogeneity is a recognized limitation in evidence synthesis and often precludes formal meta-analysis [75]. Although some studies report rapid improvement in redox balance and coagulation parameters after bariatric surgery, others demonstrate persistent abnormalities, particularly in the context of micronutrient deficiencies, underscoring the limited consistency and indirectness of the available evidence [76].

### 5.3. Hemostatic Remodeling and Persistent Thrombotic Risk

MBS leads to a rapid decline in fibrinogen levels, platelet activation markers, and PAI-1 concentrations within 3–6 months postoperatively. Fibrinogen and PAI-1 levels commonly decline during the early postoperative period, reflecting improvements in inflammatory and endothelial status after metabolic bariatric surgery [16,29,31]. These improvements reflect the resolution of the prothrombotic milieu associated with visceral adiposity and chronic inflammation [13,15,24]. However, several studies indicate that normalization of fibrinolytic activity and platelet reactivity may lag behind metabolic recovery, particularly in patients with persistent insulin resistance or low-grade inflammation [12,16,29].

Elevated postoperative D-dimer and vWF levels have been observed in certain cohorts with slower weight loss or nutritional deficits, suggesting ongoing endothelial activation despite reductions in systemic inflammatory markers. Meta-analytic studies indicate a postoperative decline in D-dimer levels, although values frequently remain above normal ranges, especially in patients with micronutrient deficiencies or incomplete weight loss [15,16,25,26].

Furthermore, certain individuals remain at risk of VTE even after clinically relevant weight loss, emphasizing that obesity-related hemostatic dysregulation is not entirely reversible [4,5,23,25]. Recent pooled analyses report postoperative VTE incidence between 0.3 and 0.5% (95% CI 0.27–0.42), with pulmonary embolism in 0.17% and deep-vein thrombosis in 0.16% of cases. The risk is notably higher after RYGB compared to SG (OR 1.7 [95% CI 1.2–2.5]) and among patients with prolonged immobilization [25,26,27].

These observations support the concept that oxidative stress and hemostatic disturbances are interdependent phenomena—both driven by inflammatory, metabolic, and nutritional factors that modulate endothelial function and coagulation pathways [7,9,16,24,55].

Collectively, these data suggest that postoperative hemostatic remodeling is multifactorial, reflecting not only surgical weight reduction but also redox status, micronutrient balance, and systemic inflammation. Persistent endothelial activation and subclinical oxidative stress may therefore sustain a residual prothrombotic state in a subset of patients, particularly those with micronutrient deficiencies or impaired antioxidant defense mechanisms [9,30,31,32,54].

### 5.4. Micronutrient Deficiencies and Redox–Hemostatic Interactions

Postoperative nutritional deficiencies—particularly of vitamins K, B12, folate, and trace elements (selenium, zinc, copper)—represent critical determinants of both oxidative and hemostatic homeostasis following MBS [6,19,32,38,46]. Vitamin K plays a central role in the γ-carboxylation of coagulation factors II, VII, IX, and X; its deficiency, frequently observed after malabsorptive procedures such as RYGB, predisposes to bleeding and prolongation of PT. Postoperative vitamin K deficiency is reported in a notable proportion of patients after RYGB and may be accompanied by prolongation of PT/INR values, reflecting impaired γ-carboxylation of vitamin K-dependent clotting factors [6,19,40]. Furthermore, suboptimal vitamin K status may also contribute to impaired vascular calcification control and endothelial dysfunction, indirectly affecting thrombotic risk [19,22].

Deficiencies in vitamins B12 and folate are equally important, as they lead to hyperhomocysteinemia—a condition linked to oxidative stress, endothelial injury, and increased platelet activation [17,20]. Elevated homocysteine levels promote superoxide generation, reduce NO bioavailability, and induce procoagulant changes in the endothelium. Postoperative monitoring of homocysteine and methylmalonic acid, combined with early supplementation of B-complex vitamins, is therefore essential to mitigate long-term thrombotic complications [20,46].

Trace elements act as cofactors for major antioxidant enzymes, linking nutritional status directly to redox and hemostatic regulation. Selenium is required for GPx activity, zinc and copper are indispensable for SOD, and magnesium contributes to platelet stability and vascular tone [32,48,49]. Deficiency of these elements disrupts antioxidant defense, enhances oxidative modification of lipoproteins and fibrinogen, and may impair platelet aggregation [47,48,49]. Studies have shown postoperative declines in circulating levels of selenium, zinc, and copper, particularly after RYGB, emphasizing the need for early biochemical assessment and lifelong supplementation [32,48,50].

Clinical guidelines from major societies, including ASMBS, ESPEN, and BOMSS (British Obesity and Metabolic Surgery Society), highlight the necessity of individualized micronutrient replacement to prevent oxidative and coagulation-related complications [38,39,46,51]. Regular biochemical monitoring of vitamins and trace elements is recommended at 3, 6, and 12 months postoperatively and annually thereafter, with higher frequency in malabsorptive procedures or patients with persistent inflammation [39,46,51,52].

Taken together, these findings underscore that micronutrient deficiencies are not merely metabolic side effects of bariatric surgery but play a pivotal pathophysiological role in maintaining redox–hemostatic balance. Failure to correct them may perpetuate endothelial dysfunction, promote thromboinflammation, and counteract the vascular benefits of weight loss [6,9,20,32,42,46,47,48,49,54].

### 5.5. Integrative Mechanisms Linking Oxidative and Hemostatic Pathways

The interplay between oxidative stress and hemostasis is complex and bidirectional, involving multiple molecular feedback loops that connect endothelial, platelet, and coagulation pathways [7,9,16,24,55]. ROS generated during chronic inflammation promote oxidative modification of endothelial membranes, reduce NO bioavailability, and enhance expression of prothrombotic mediators such as TF and vWF [13,24,36]. These processes contribute to platelet activation and aggregation, initiating a cycle of vascular oxidative injury and hypercoagulability.

Excessive ROS production also affects fibrinolytic balance by increasing PAI-1 and reducing thrombomodulin expression, thereby impairing fibrin degradation and sustaining a procoagulant phenotype [16,29,55]. Conversely, activation of the coagulation cascade itself can generate additional ROS through thrombin-mediated NADPH oxidase activity, reinforcing endothelial dysfunction and platelet activation [36,55]. This self-amplifying mechanism links oxidative stress and thrombosis in a reciprocal manner, explaining why postoperative oxidative imbalance can prolong hemostatic recovery even in patients with substantial metabolic improvement [7,9,31,54].

Following MBS, weight loss and the associated reduction in systemic inflammation decrease oxidative burden and restore antioxidant capacity, leading to improved endothelial reactivity and reduced PAI-1 and fibrinogen levels [7,15,29,31]. Nonetheless, incomplete normalization of oxidative pathways—particularly in patients with persistent micronutrient deficiencies or chronic inflammation—may perpetuate low-grade endothelial activation, with elevated vWF or D-dimer may persist in certain cohorts [9,15,16,25].

These findings collectively suggest that MBS acts as a partial “biological reset” of the oxidative–hemostatic axis: it alleviates but does not entirely eliminate the prothrombotic effects of obesity-induced redox imbalance. Continued research integrating redox, coagulation, and endothelial biomarkers is necessary to clarify the molecular determinants of residual thrombogenicity after bariatric surgery [7,9,16,24,55].

### 5.6. Clinical Implications for Thromboprophylaxis and Monitoring

Recognition of the redox–hemostatic axis after MBS has direct clinical relevance for optimizing perioperative prevention and monitoring strategies. Despite major improvements in metabolic and vascular function, VTE remains one of the most serious postoperative complications.

A recent meta-analysis of 87 studies including 2.7 million patients reported a cumulative incidence of postoperative VTE of 0.34% (95% CI 0.27–0.42), with pulmonary embolism in 0.17% and deep-vein thrombosis in 0.16% of cases [25]. Population-based data from Froehling et al. indicated an adjusted hazard ratio (HR) 2.8 [95% CI 1.9–4.2] for VTE within 30 days after surgery compared with non-surgical controls [26]. Risk is markedly higher after RYGB compared with SG (odds ratio 1.7 [95% CI 1.2–2.5]) and among patients with prolonged immobility or BMI > 50 kg/m^2^ [23,25,27].

Given the bidirectional relationship between oxidative stress and coagulation, redox imbalance may represent an emerging component of postoperative risk stratification. Persistent oxidative and endothelial activation—reflected by elevated D-dimer or vWF—can signify delayed hemostatic recovery and should prompt closer follow-up [7,9,16,24].

Routine postoperative laboratory evaluation should include complete blood count, PT/INR, fibrinogen, D-dimer, and platelet indices, complemented by periodic micronutrient screening (vitamins K, B12, folate, and trace elements) to address the nutritional determinants of redox and coagulative homeostasis [6,32,39,42,46,51].

Integrating biochemical, nutritional, and clinical parameters enables a personalized approach to VTE prevention, balancing thrombosis and bleeding risk while supporting vascular recovery after MBS [4,5,23,27,34,35,41].

### 5.7. Future Directions and Research Gaps

Despite substantial progress in understanding the metabolic and vascular effects of MBS, the precise molecular mechanisms linking oxidative stress and hemostatic remodeling remain incompletely defined [7,9,16,24,31,54,55]. Current evidence is largely derived from small observational cohorts and heterogeneous biomarker assessments, limiting the ability to establish causal relationships. Future studies should prioritize prospective, adequately powered randomized controlled trials (RCTs) that systematically evaluate both redox and coagulation parameters in parallel, integrating metabolic, inflammatory, and nutritional factors.

A major limitation of existing literature is the lack of standardized redox biomarker panels. Most studies employ single indicators—such as MDA, oxLDL, or total antioxidant capacity—without accounting for enzyme-based antioxidant systems (SOD, GPx, catalase). Reported effect sizes for oxidative stress reduction after metabolic bariatric surgery vary substantially across studies, reflecting methodological heterogeneity, differences in biomarker selection, and inconsistent assay calibration [7,9,31,54]. Establishing validated composite indices of oxidative and endothelial status could enhance comparability and predictive accuracy for thrombotic or bleeding complications [9,31,54]. In addition, inclusion of advanced markers such as NADPH oxidase activity, PAI-1, or circulating microRNAs regulating redox–hemostatic signaling could deepen mechanistic understanding [55].

Another critical gap concerns the impact of micronutrient and antioxidant supplementation on hemostatic outcomes. Although guidelines from ASMBS and ESPEN emphasize routine replacement, high-quality data quantifying the effects of specific micronutrients (e.g., vitamin K, selenium, zinc) on coagulation dynamics remain scarce [39,42,46,51]. Future RCTs should compare standard vs. intensified micronutrient protocols with integrated monitoring of oxidative stress markers, endothelial activation, and platelet function.

Finally, research should explore multimodal predictive models combining clinical scores (e.g., modified Caprini score), oxidative biomarkers, and micronutrient profiles to identify patients at persistent thrombotic risk despite metabolic improvement [37,54,55]. Such integrated algorithms could refine postoperative monitoring, optimize thromboprophylaxis duration, and support individualized long-term care strategies.

Collectively, these directions highlight the need for a translational framework that bridges biochemical insight with clinical implementation, transforming MBS follow-up from weight-centered to metabolism- and hemostasis-centered paradigms [7,9,16,24,54,55].

### 5.8. Limitations

This review has several important limitations that should be acknowledged. First, the available studies investigating coagulation and oxidative stress after MBS exhibit marked heterogeneity in design, patient selection, and follow-up duration [7,9,16,31,54]. Most are retrospective or observational, with small sample sizes and limited adjustment for potential confounders such as age, diabetes, medication use, or inflammatory comorbidities. Consequently, the comparability of reported outcomes, including changes in oxidative biomarkers or coagulation parameters, remains restricted.

Second, there is a lack of standardized laboratory assays and reference ranges for oxidative stress evaluation, leading to inconsistent quantification of biomarkers such as MDA, SOD and GPx across studies [9,31,54]. Similarly, differences in surgical technique (RYGB vs. SG) and postoperative supplementation protocols further contribute to variability in hemostatic recovery [29,32,46].

Finally, although this synthesis aimed to integrate metabolic, nutritional, and hemostatic perspectives, the exclusion of non-English studies and limited access to raw data may have led to selection bias. Future research should address these limitations by applying standardized methodology, uniform biomarker panels, and long-term multicenter follow-up to improve reproducibility and clinical translation [16,25,54].

## 6. Conclusions

MBS exerts a multidimensional effect on coagulation, oxidative balance, and vascular homeostasis. Weight reduction and metabolic improvement alleviate inflammation and oxidative stress, leading to partial restoration of endothelial and hemostatic function [7,9,15,16,24,31]. However, persistent oxidative imbalance and micronutrient deficiencies, particularly in vitamins K, B12, folate, and trace elements, may sustain a residual thrombotic or hemorrhagic risk [6,19,32,42,46].

Effective long-term management after MBS, therefore, requires an integrative approach combining metabolic control, nutritional optimization, and individualized thromboprophylaxis. Regular biochemical and redox monitoring should complement classical coagulation tests to detect subclinical dysregulation before clinical complications arise [7,9,16,34,46].

Future translational efforts should focus on the development of standardized oxidative and endothelial biomarkers, randomized studies evaluating antioxidant and micronutrient interventions, and predictive algorithms that integrate metabolic, nutritional, and hemostatic parameters [9,31,54,55].

In conclusion, maintaining hemostatic stability after bariatric surgery extends beyond surgical success—it depends on sustained metabolic balance, adequate micronutrient support, and coordinated multidisciplinary care [7,9,16,24,46,54].

## Figures and Tables

**Table 1:** Thrombotic and hemorrhagic risk factors after metabolic bariatric surgery, their underlying mechanisms, and clinical implications.

Type of Risk	Risk Factor	Mechanism/Pathophysiology	Clinical Significance	Sources
thrombotic	Morbid obesity	Chronic inflammatory state, ↑ PAI-1, ↑ fibrinogen, platelet activation	Baseline hypercoagulability before surgery	[2,13,16,24,36]
	Obstructive sleep apnea	Nocturnal hypoxemia → ↑ sympathetic activity → activation of coagulation	Increased risk of MI/CHD	[16,24,35,36]
	Immobilization after surgery	Venous blood stasis	Most common risk factor for DVT	[3,25,35,37]
	History of DVT/PE	Persistent thrombophilic predisposition	High risk of recurrence, need for prolonged prophylaxis	[5,23,25,34,35]
	Severe obesity	↑ blood volume, ↑ inflammation	Higher risk of thrombotic complications	[1,2,24,29,36]
hemorrhagic	Vitamin K deficiency after RYGB/BPD-DS	Impaired vitamin K absorption in the small intestine	Prolonged INR, bleeding tendency	[6,19,29,38,39,40]
	Pharmacoprophylaxis (LMWH)	Prolonged anticoagulation	↑ risk of postoperative bleeding	[3,5,27,34,35,41]
	B12 and folic acid deficiencies	Impaired erythropoiesis, thrombocytopathy	Bleeding from stapler lines, bruising	[17,20,29,33,39,42,43]
	Revision procedures	Larger staple area, longer operating time	↑ risk of early bleeding	[3,4,23,25,41]

Abbreviations: BPD-DS; Biliopancreatic Diversion with Duodenal Switch; RYGB, Roux-en-Y gastric bypass; LMWH, low-molecular-weight heparin; DVT, deep vein thrombosis; MI, myocardial Infarction; CHD, coronary heart disease; PE, pulmonary embolism; INR, international normalized ratio; PAI-1, plasminogen activator inhibitor-1.

## Data Availability

No new data were created or analyzed in this study. Data sharing is not applicable to this article.

## Supplementary Materials

The following supporting information can be downloaded at: https://www.mdpi.com/article/10.3390/antiox15010124/s1, Table S1: Characteristics on Included Studies, Table S2: Risk of Bias Assessment of the Included Studies (NOS/AXIS), Table S3: PRISMA 2020 Checklist for the Present Systematic Review, Table S4: Oxidative Stress Markers in Included Studies, Table S5: Hemostatic and Coagulation Markers in Included Studies, Table S6: Micronutrient Status and Trace Elements in Includes Studies.

## Abbreviations

The following abbreviations are used in this manuscript:

AACE	American Association of Clinical Endocrinologists
AXIS	Appraisal tool for Cross-Sectional Studies
ASA	American Society of Anesthesiologists
ASMBS	American Society for Metabolic and Bariatric Surgery
BMI	Body Mass Index
BOMSS	British Obesity and Metabolic Surgery Society
BPD-DS	Biliopancreatic Diversion with Duodenal Switch
CHD	Coronary Heart Disease
CI	Confidence Interval
CRP	C-reactive Protein
DOAC	Direct Oral Anticoagulant
DVT	Deep Vein Thrombosis
EASO	European Association for the Study of Obesity
ERAS	Enhanced Recovery After Surgery
ESPEN	European Society for Clinical Nutrition and Metabolism
ESVS	European Society for Vascular Surgery
GPx	Glutathione Peroxidase
GRADE	Grading of Recommendations, Assessment, Development and Evaluations
HR	Hazard Ratio
IL-6	Interleukin-6
INR	International Normalized Ratio
LMWH	Low-Molecular-Weight Heparin
MBSAQIP	Metabolic and Bariatric Surgery Accreditation and Quality Improvement Program
MBS	Metabolic Bariatric Surgery
MDA	Malondialdehyde
MI	Myocardial Infarction
MUST	Malnutrition Universal Screening Tool
NO	Nitric Oxide
NOS	Newcastle–Ottawa Scale
OAGB	One-Anastomosis Gastric Bypass
OR	Odds Ratio
OSA	Obstructive Sleep Apnea
oxLDL	Oxidized Low-Density Lipoprotein
PAI-1	Plasminogen Activator Inhibitor-1
PE	Pulmonary Embolism
PK	Pharmacokinetics
PROSPERO	International Prospective Register of Systematic Reviews
PT	Prothrombin Time
RCT	Randomized Controlled Trial
ROS	Reactive Oxygen Species
RYGB	Roux-en-Y Gastric Bypass
SG	Sleeve Gastrectomy
SGA	Subjective Global Assessment
SIBO	Small Intestinal Bacterial Overgrowth
SOD	Superoxide Dismutase
TNF- α	Tumor Necrosis Factor α
VKA	Vitamin K Antagonist
vWF	von Willebrand Factor
VTE	Venous Thromboembolism
