# Supplementary material for: Oxidative Stress, Micronutrient Deficiencies and Coagulation Disorders After Bariatric Surgery: A Systematic Review
Source: Antioxidants (Basel). 2026 Jan 18;15(1):124. doi: 10.3390/antiox15010124 (PMC12837164; doi:10.3390/antiox15010124)
Supplement: Supplementary file 1 [file antioxidants-15-00124-s001.zip › Table S1.pdf]

Table S1. Characteristics of Included Studies (n = 21)

This table lists all studies included in the qualitative synthesis. Extracted automatically from Table S2.

Columns follow MDPI standards. Data fields will be populated in the next step.

Author (Year)	Country	Study Design	n	Procedure	Outcomes	Follow-up	Key Findings
Ion et al. (2025)	—	Prospective cohort	NR	Bariatric surgery (mixed, not specified)	Oxidative stress / hemostatic markers	Up to 12 months (NR exact)	Improved redox balance and antioxidant response after bariatric surgery.
Wroblewski et al. (2016)	Poland	Prospective cohort	NR	Mixed bariatric/endoscopic therapies	Hormones / inflammatory markers	Short- to mid-term (NR exact)	Weight loss associated with favorable changes in hormonal profile and inflammation.
Vázquez et al. (2005)	Spain	Cohort	NR	Bariatric surgery (unspecified, likely	Inflammation / endothelial function	NR	Weight loss improved endothelial

				RYGB)			function and reduced inflammatory markers.
Sachan et al. (2022)	India	Cohort	Small sample (NR exact)	Bariatric surgery (likely SG predominance)	Adipokines / inflammatory markers	Early postoperative period	Demonstrated early postoperative changes in adipocytokines and inflammation after bariatric surgery.
Jensen et al. (2025)	Denmark	Prospective cohort	NR	RYGB and SG	Inflammatory markers / hemostatic markers	12 months	Longitudinal reduction in CRP and IL-6 with differences between procedures.
Poglitsch et al. (2020)	Austria	Retrospective cohort	NR	Laparoscopic RYGB	Homocysteine / vitamin B12 / folate	Early and long-term follow-up	Homocysteine increased early after RYGB and decreased below

							baseline at long-term follow-up.
Leslie et al. (2025)	USA	Database cohort (multicenter)	Large administrative dataset	Various bariatric procedures	Venous thromboembolism	Up to 30 days and beyond (registry-based)	Identified predictors and trends in postoperative VTE after bariatric surgery.
Froehling et al. (2013)	USA	Population-based cohort	NR	Bariatric surgery (mixed procedures)	VTE incidence	30 days and longer-term	Showed low but clinically important incidence of VTE after bariatric surgery compared with non-surgical controls.
Ali et al. (2024)	—	Retrospective cohort with machine learning	NR	Roux-en-Y gastric bypass	Thromboembolic risk prediction	Short-term postoperative	Machine learning models predicted short-term VTE risk after RYGB using clinical

							variables.
Lupoli et al. (2015)	Italy	Comparative cohort	NR	RYGB vs SG	Coagulation and fibrinolytic markers	6–12 months (NR exact)	Both procedures improved hypercoagulable profiles with some differences between RYGB and SG.
Şimşek et al. (2023)	Türkiye	Prospective cohort	NR	Sleeve gastrectomy	Oxidative stress markers	12 months	Showed gradual improvement in oxidative stress indices over one year after SG.
Carmona-Maurici et al. (2020)	Spain	Cohort	NR	Bariatric surgery (mixed; often RYGB/SG)	Oxidative stress and atheroma status	NR	Bariatric surgery improved oxidative stress markers; presence of atheroma modulated the

							response.
Hierons et al. (2023)	UK	Cross-sectional	NR	Patients before and after bariatric surgery	Magnesium, zinc, copper, selenium	Cross-sectional (single timepoints)	Demonstrated frequent trace element abnormalities in obese patients undergoing bariatric surgery.
Chin et al. (2024)	USA	Before–after cohort	NR	Bariatric procedures with standardized heparin protocol	Postoperative bleeding / VTE	Early postoperative period	Implementation of a standardized heparin protocol reduced VTE without excess major bleeding.
Rabl et al. (2011)	USA	Retrospective cohort	NR	Roux-en-Y gastric bypass	Early and late postoperative bleeding	Early and late postoperative	Characterized sources and timing of early and late bleeding after

							RYGB and discussed tailored management.
Rottenstreich et al. (2018)	Israel	Cohort (pharmacokinetic)	NR	Bariatric surgery (RYGB/SG)	Direct oral anticoagulant (DOAC) levels	Postoperative steady-state assessments	Found variable DOAC levels after bariatric surgery, suggesting need for caution and monitoring.
Papamargaritis et al. (2015)	UK	Cohort	NR	Bariatric surgery with supplementation	Copper, selenium, zinc	Postoperative follow-up (NR exact)	Documented deficiencies in copper, selenium and zinc despite standard multivitamin supplementation.
Ramos-Luzardo et al. (2025)	Spain	Prospective cohort	NR	RYGB or SG (obese cohort after BS)	Serum inorganic elements	Postoperative (NR exact)	Showed changes in serum inorganic

							element concentrations after bariatric surgery, with implications for oxidative stress.
Vieira de Sousa et al. (2024)	Portugal	Comparative cohort	NR	RYGB vs SG	Micronutrient deficiencies	Postoperative follow-up (NR exact)	Compared prevalence of nutritional deficiencies between RYGB and SG patients.
Henning et al. (2022)	Germany	Cross-sectional	NR	Candidates for RYGB/SG	Preoperative micronutrient status	Preoperative only	Reported high prevalence of micronutrient abnormalities before bariatric surgery.
Jans et al. (2014)	Belgium	Prospective cohort	NR	Pregnant women after	Vitamin K1 levels	Pregnancy course	Showed that vitamin

				bariatric surgery			K1 status requires monitoring in pregnancies after bariatric surgery.
Menser et al. (2020)	USA	Cohort	NR	Bariatric surgery (RYGB/SG)	Laboratory monitoring frequency and yield	Postoperative follow-up (NR exact)	Suggested that some routine postoperative lab tests may be redundant while others are essential for detecting deficiencies.
