# Supplementary material for: Oxidative Stress, Micronutrient Deficiencies and Coagulation Disorders After Bariatric Surgery: A Systematic Review
Source: Antioxidants (Basel). 2026 Jan 18;15(1):124. doi: 10.3390/antiox15010124 (PMC12837164; doi:10.3390/antiox15010124)
Supplement: Supplementary file 1 [file antioxidants-15-00124-s001.zip › Table S2.pdf]

Table S2. Risk of Bias Assessment of the Included Studies (NOS / AXIS)

This table summarizes the risk of bias assessment for all studies cited in the manuscript. Cohort and case–control studies were evaluated using the Newcastle–Ottawa Scale (NOS), while cross-sectional studies were evaluated using the AXIS tool. Reviews, guidelines, and commentaries were marked as 'Not applicable'. Two reviewers (K.G. and A.Ł.) independently assessed risk of bias; disagreements were resolved by consensus.

Cut-offs:
• NOS: 7–9 = Low risk; 5–6 = Moderate; <5 = High
• AXIS: 17–20 = Low; 12–16 = Moderate; <12 = High

Author (Year)	Study Design	Tool	Score	Risk Level	Key Limitations	Included in Synthesis
Ion et al. (2025)	Prospective cohort	NOS	8/9	Low	Single-center; biomarker-only endpoints	Yes
Wroblewski et al. (2016)	Prospective cohort	NOS	7/9	Moderate	Single-center; no randomization	Yes
Vázquez et al. (2005)	Cohort	NOS	7/9	Moderate	Older endothelial assays	Yes
Sachan et al. (2022)	Cohort	NOS	6/9	Moderate	Small sample; short follow-up	Yes
Jensen et al. (2025)	Prospective cohort	NOS	9/9	Low	Procedure heterogeneity	Yes
Poglitsch et al. (2020)	Retrospective cohort	NOS	7/9	Moderate	Retrospective; confounding	Yes

Leslie et al. (2025)	Database cohort	NOS	9/9	Low	Administrative dataset	Yes
Froehling et al. (2013)	Population cohort	NOS	9/9	Low	Population-level; limited biomarkers	Yes
Ali et al. (2024)	Retrospective ML cohort	NOS	7/9	Moderate	Modeling bias; retrospective	Yes
Lupoli et al. (2015)	Comparative cohort	NOS	8/9	Low	Different procedures	Yes
Şimşek et al. (2023)	Prospective cohort	NOS	8/9	Low	Limited oxidative markers	Yes
Carmona-Maurici et al. (2020)	Cohort	NOS	8/9	Low	Mid-size sample	Yes
Hierons et al. (2023)	Cross-sectional	AXIS	17/20	Low	Single timepoint assessment	Yes
Chin et al. (2024)	Before–after cohort	NOS	7/9	Moderate	Protocol-only outcomes	Yes
Rabl et al. (2011)	Retrospective cohort	NOS	7/9	Moderate	Older data	Yes
Rottenstreich et al. (2018)	Cohort (PK DOAC)	NOS	7/9	Moderate	Small sample; PK variability	Yes
Papamargaritis et al. (2015)	Cohort	NOS	8/9	Low	Trace elements only	Yes
Ramos-Luzardo et al. (2025)	Prospective cohort	NOS	8/9	Low	Biomonitoring; limited clinical endpoints	Yes
Vieira de Sousa et al.	Comparative	NOS	8/9	Low	RYGB vs SG	Yes

(2024)	cohort				only	
Henning et al. (2022)	Cross-sectional	AXIS	16/20	Moderate	Pre-op analysis only	Yes
Jans et al. (2014)	Prospective cohort	NOS	7/9	Moderate	Pregnancy cohort	Yes
Menser et al. (2020)	Cohort	NOS	6/9	Moderate	Limited confounder control	Yes
Blüher et al. (2019)	Review	N/A	–	–	Not primary data	No
Stenberg et al. (2022)	Guideline	N/A	–	–	Not primary data	No
Mandour et al. (2024)	Review	N/A	–	–	Not primary data	No
Sherf-Dagan et al. (2019)	Review	N/A	–	–	Not primary data	No
Ribeiro et al. (2024)	Review	N/A	–	–	Not primary data	No
Vincent & Taylor (2006)	Review	N/A	–	–	Not primary data	No
Hotamisligil (2006)	Review	N/A	–	–	Not primary data	No
Bladbjerg et al. (2020)	Review	N/A	–	–	Not primary data	No
Aaseth & Alexander (2023)	Review	N/A	–	–	Not primary data	No
Shearer & Newman	Review	N/A	–	–	Not primary data	No

(2008)						
Meshram et al. (2023)	Review	N/A	–	–	Not primary data	No
Fan & Pedersen (2021)	Review	N/A	–	–	Not primary data	No
Yan et al. (2022)	Review	N/A	–	–	Not primary data	No
Zawadzka et al. (2025)	Review	N/A	–	–	Not primary data	No
El Ansari et al. (2024)	Review	N/A	–	–	Not primary data	No
Almarshad et al. (2020)	Review	N/A	–	–	Not primary data	No
Sorodoc et al. (2024)	Review	N/A	–	–	Not primary data	No
Aminian et al. (2022)	Guideline	N/A	–	–	Not primary data	No
Kakkos et al. (2021)	Guideline	N/A	–	–	Not primary data	No
Giannopoulos et al. (2023)	Review	N/A	–	–	Not primary data	No
Gupta et al. (2018)	Review	N/A	–	–	Not primary data	No
H. AJ et al.	Review	N/A	–	–	Not primary data	No
Busetto et al. (2017)	Guideline	N/A	–	–	Not primary data	No
Caprini (2005)	Review	N/A	–	–	Not primary	No

					data	
Steenackers et al. (2025)	Review	N/A	–	–	Not primary data	No
Nutescu et al. (2009)	Review	N/A	–	–	Not primary data	No
Freeman et al. (2010)	Review	N/A	–	–	Not primary data	No
Lupoli et al. (2017)	Review	N/A	–	–	Not primary data	No
Parrott et al. (2017)	Guideline	N/A	–	–	Not primary data	No
Wróblewski et al. (2024)	Review	N/A	–	–	Not primary data	No
Choi et al. (2018)	Review	N/A	–	–	Not primary data	No
Pradel-Mora et al. (2024)	Review	N/A	–	–	Not primary data	No
Li et al. (2024)	Review	N/A	–	–	Not primary data	No
Mechanick et al. (2020)	Guideline	N/A	–	–	Not primary data	No
Aminian et al. (2017)	Review	N/A	–	–	Not primary data	No
