# Supplementary material for: Oxidative Stress, Micronutrient Deficiencies and Coagulation Disorders After Bariatric Surgery: A Systematic Review
Source: Antioxidants (Basel). 2026 Jan 18;15(1):124. doi: 10.3390/antiox15010124 (PMC12837164; doi:10.3390/antiox15010124)
Supplement: Supplementary file 1 [file antioxidants-15-00124-s001.zip › Table S3 PRISMA 2020 Checklist.pdf]

Table S2. PRISMA 2020 Checklist for the Present Systematic Review

Section and Topic	Item #	PRISMA 2020 Checklist item	Location in Manuscript
TITLE	1	Identify the report as a systematic review.	Title page
ABSTRACT	2	Provide a structured summary including background, objectives, data sources, study eligibility criteria, participants, interventions, and main results.	Abstract
INTRODUCTION	3	Describe the rationale for the review in the context of existing knowledge.	Section 1
	4	Provide an explicit statement of the objectives or questions being addressed.	Section 1
METHODS	5	Specify inclusion and exclusion criteria, including study design, population, and outcomes of interest.	Section 2.2
	6	Describe all information sources (e.g., databases with dates of coverage) and the date last searched.	Section 2.1
	7	Present the full electronic search strategy for at least one database, including all search terms and limits used.	Section 2.1 and Supplementary File (search codes)
	8	Specify the methods used to select studies (screening, eligibility, inclusion).	Section 2.3
	9	Describe the methods	Section 2.3

		of data collection and extraction.	
	10	List and define all variables for which data were sought (e.g., outcomes, study characteristics).	Section 2.3
	11	Describe any methods used to assess risk of bias in included studies.	Section 2.4
	12	Specify any methods used to assess certainty or confidence in the body of evidence.	Section 2.5
	13	Describe any methods used to synthesize results and assess heterogeneity.	Section 2.5
	14	Explain any methods used to explore publication bias or small-study effects.	Section 2.5
	15	Describe any sensitivity analyses conducted.	Section 2.4 and 2.5
RESULTS	16	Describe the results of the search and selection process, ideally using a flow diagram.	Section 3 and Figure 1
	17	Provide characteristics of included studies (e.g., study size, PICOS, follow-up).	Section 3.1 and Table 1
	18	Present results for each outcome, including summary data and key findings.	Sections 3.2–3.4
	19	Report results of syntheses, including consistency across studies.	Section 3.4
	20	Present assessments of risk of bias for each included study.	Section 2.4 and Table S2

DISCUSSION	21	Summarize main findings and discuss relevance to existing evidence.	Section 4
	22	Discuss strengths, limitations, and potential biases in the review process.	Section 4.3
	23	Provide conclusions that relate to the review objectives and implications for future research.	Section 5
OTHER INFORMATION	24	Describe registration information and protocol availability.	Not registered in PROSPERO; this review focused on an integrative narrative synthesis rather than an intervention-specific meta-analysis (Section 2.1).
	25	Describe sources of support, funding, and role of funders.	Funding statement
	26	Declare any competing interests.	Conflicts of Interest section
	27	Report availability of data, code, and materials.	Data Availability Statement
