# Supplementary material for: Oxidative Stress, Micronutrient Deficiencies and Coagulation Disorders After Bariatric Surgery: A Systematic Review
Source: Antioxidants (Basel). 2026 Jan 18;15(1):124. doi: 10.3390/antiox15010124 (PMC12837164; doi:10.3390/antiox15010124)
Supplement: Supplementary file 1 [file antioxidants-15-00124-s001.zip › Table S4 OxidativeStress.pdf]

Table S4. Oxidative Stress Markers in Included Studies

This table summarizes oxidative stress biomarker outcomes from primary studies included in the review. Values are presented as directional changes (↑ increase, ↓ decrease, ↔ no change) to avoid numerical fabrication when exact quantitative results are not available in the extracted text. Markers include MDA (malondialdehyde), TAC (total antioxidant capacity), SOD (superoxide dismutase), GPx (glutathione peroxidase), and CAT (catalase).

Author (Year)	Procedure	Markers Assessed	Pre-op	Post-op	Direction of Change	Key Notes
Şimşek et al. (2023)	SG	MDA, TAC, SOD, GPx	High oxidative stress baseline	Improved at 12 months	MDA ↓, TAC ↑, SOD ↑, GPx ↑	Consistent postoperative improvement in OS profile.
Ion et al. (2025)	Mixed BS	MDA, TAC, total ROS	Elevated oxidative stress	Reduced oxidative stress at follow-up	MDA ↓, TAC ↑	General improvement after weight loss.
Carmona-Maurici et al. (2020)	BS (mixed)	Oxidative stress index, atheroma markers	Variable by atheroma status	Improved in non-atheroma patients	OS ↓	Atheroma presence attenuated improvement.
Ramos-Luzardo et al. (2025)	RYGB / SG	Trace elements related to OS (Zn, Cu, Se)	Baseline alterations	Improved micronutrient-related OS indices	Zn ↑, Se ↑	Element restoration parallels reduced oxidative imbalance.
Papamargaritis et al. (2015)	Bariatric surgery + supplementation	Zn, Cu, Se	Frequent deficiencies	Variable normalization	Zn ↑/↔, Se ↑	Responses depend on adherence to supplementation.
