# Supplementary material for: Oxidative Stress, Micronutrient Deficiencies and Coagulation Disorders After Bariatric Surgery: A Systematic Review
Source: Antioxidants (Basel). 2026 Jan 18;15(1):124. doi: 10.3390/antiox15010124 (PMC12837164; doi:10.3390/antiox15010124)
Supplement: Supplementary file 1 [file antioxidants-15-00124-s001.zip › Table S5 Hemostasis.pdf]

Table S5. Hemostatic and Coagulation Markers in Included Studies

This table summarizes coagulation and hemostatic outcomes from primary studies included in the review. Values are expressed as directional trends (↑ increase, ↓ decrease, ↔ no change) based on reported findings, without fabricating numerical data. Markers include D-dimer, fibrinogen, PAI-1, INR/PT, APTT, platelet indices, and homocysteine (HCY), as well as pharmacokinetic findings for oral anticoagulants (DOACs).

Author (Year)	Procedure	Markers Assessed	Pre-op	Post-op	Direction of Change	Key Notes
Lupoli et al. (2015)	RYGB vs SG	Fibrinogen, D-dimer, PAI-1, PT/INR	Elevated coagulation activation	Improved post-op	Fibrinogen ↓, D-dimer ↓, PAI-1 ↓	Greater improvements observed after RYGB.
Poglitsch et al. (2020)	RYGB	Homocysteine (HCY), B12, folate	Baseline HCY normal/variable	Transient increase, then decrease	HCY ↑ early, ↓ long-term	Homocysteine followed a biphasic pattern post-RYGB.
Jensen et al. (2025)	RYGB / SG	CRP, IL-6, endothelial markers, D-dimer	High inflammatory/coag profile	Improved during follow-up	D-dimer ↓, inflammation ↓	Inflammatory and prothrombotic markers decreased over time.
Rottenstreich et al. (2018)	RYGB / SG	DOAC plasma levels (PK)	Standard preoperative PK	Altered absorption post-op	Variable/↓	PK variability suggests need for anti-Xa monitoring.
Chin et al. (2024)	Bariatric surgery (heparin protocol)	Bleeding rates, VTE events	Baseline protocol variable	Improved outcomes	VTE ↓, major bleeding ↔	Standardized heparin protocol reduced VTE without increasing bleeding.
Rabl et al. (2011)	RYGB	Postoperative bleeding (early/late)	Baseline risk low	Varied by site/time	Bleeding risk ↑ early	Staple-line bleeding predominant in early period.
Froehling et al. (2013)	Mixed BS	VTE incidence	Baseline population risk	Post-BS risk low but present	VTE ↓ vs obese controls	Population-based reduction but nonzero postoperative risk.
Leslie et al. (2025)	Mixed BS	VTE risk factors	Baseline risk elevated in severe obesity	Risk stratified by predictors	VTE ↓ overall with optimized pathways	Identified modifiable and non-modifiable VTE predictors.
