# Supplementary material for: Oxidative Stress, Micronutrient Deficiencies and Coagulation Disorders After Bariatric Surgery: A Systematic Review
Source: Antioxidants (Basel). 2026 Jan 18;15(1):124. doi: 10.3390/antiox15010124 (PMC12837164; doi:10.3390/antiox15010124)
Supplement: Supplementary file 1 [file antioxidants-15-00124-s001.zip › Table S6 Micronutrients.pdf]

Table S6. Micronutrient Status and Trace Elements in Included Studies

This table summarizes micronutrient and trace-element outcomes from primary studies included in the review. Directional changes (↑ increase, ↓ decrease, ↔ no change) are used when exact numerical values were not available. Markers include vitamin K1, vitamin B12, folate, zinc (Zn), copper (Cu), selenium (Se), magnesium (Mg), and other biochemically relevant elements.

Author (Year)	Procedure	Micronutrients Assessed	Pre-op Status	Post-op Status	Direction of Change	Key Notes
Hierons et al. (2023)	Mixed BS	Zn, Cu, Se, Mg	Common deficiencies pre-op	Persisting or partial correction	Zn ↓/↔, Cu ↓/↔, Se ↓/↔	Trace element abnormalities common both before and after BS.
Papamargaritis et al. (2015)	Mixed BS + supplementation	Zn, Cu, Se	Frequent deficiencies	Variable normalization after supplementation	Zn ↑/↔, Cu ↔, Se ↑	Supplementation improved some but not all deficiencies.
Ramos-Luzardo et al. (2025)	RYGB / SG	Zn, Cu, Se, other inorganic elements	Altered baseline inorganic elements	Improvement in selected elements	Zn ↑, Se ↑, Cu ↔/↑	Element restoration associated with improved oxidative balance.
Jans et al. (2014)	Post-BS pregnancy cohort	Vitamin K1	Variable K1 levels	Potential risk of deficiency during pregnancy	K1 ↓/↔	Highlights need for vitamin K monitoring in pregnancy after BS.
Menser et al. (2020)	RYGB / SG	B12, folate, iron (indirect)	Common deficiencies	Monitoring identified ongoing deficits	B12 ↓/↔, folate ↓/↔	Routine labs may miss subtle deficiencies.
